# *N*,*N*-Dimethyl-*p*-toluidine: MAK Value Documentation – Translation of the German version from 2022

**DOI:** 10.34865/mb9997e10_1or

**Published:** 2025-03-31

**Authors:** Andrea Hartwig, MAK Commission

**Affiliations:** 1 Institute of Applied Biosciences. Department of Food Chemistry and Toxicology. Karlsruhe Institute of Technology (KIT) Adenauerring 20a, Building 50.41 76131 Karlsruhe Germany; 2 Permanent Senate Commission for the Investigation of Health Hazards of Chemical Compounds in the Work Area. Deutsche Forschungsgemeinschaft, Kennedyallee 40, 53175 Bonn, Germany. Further information: Permanent Senate Commission for the Investigation of Health Hazards of Chemical Compounds in the Work Area | DFG

**Keywords:** N,N-dimethyl-p-toluidine, carcinogenicity, skin absorption, germ cell mutagenicity, irritation, metabolism, toxicity, methaemoglobinaemia, liver

## Abstract

The German Commission for the Investigation of Health Hazards of Chemical Compounds in the Work Area (MAK Commission) has evaluated the occupational exposure limit value (maximum concentration at the workplace, MAK value) of *N*,*N*-dimethyl-*p*-toluidine [99-97-8] considering all toxicological end points. Relevant studies were identified from a literature search. *N*,*N*-Dimethyl-*p*-toluidine led to an increased incidence of liver adenomas and carcinomas in mice at 6 mg/kg body weight and day and in rats at 60 mg/kg body weight and day in a chronic gavage study. Additional tumours were observed in male rats in the transitional epithelium of the nose and in female rats in the lungs and the forestomach. On the basis of these effects, *N*,*N*-dimethyl-*p*-toluidine has been classified in Carcinogen Category 2. *N*,*N*-Dimethyl-*p*-toluidine induces mutagenic, aneugenic and clastogenic effects in vitro. In vivo, *N*,*N*-dimethyl-*p*-toluidine was found to cause DNA damage in the livers of rats and mice. As studies for germ cell mutagenicity are not available, *N*,*N*-dimethyl-*p*-toluidine has been classified in Germ Cell Mutagenicity Category 3 B. According to skin absorption models, percutaneous absorption is expected to contribute significantly to systemic toxicity. Therefore, *N*,*N*-dimethyl-*p*-toluidine has been designated with “H”. A sensitizing potential is not expected from the data available.

**Table:** 

**MAK value**	**–**
**Peak limitation**	**–**
	
**Absorption through the skin (2021)**	**H**
**Sensitization**	**–**
**Carcinogenicity (2021)**	**Category 2**
**Prenatal toxicity**	**–**
**Germ cell mutagenicity (2021)**	**Category 3 B**
	
**BAT value**	**–**
	
Synonyms	4-(dimethylamino)toluene4-methyl-*N*,*N*-dimethylaniline
Chemical name (IUPAC)	*N*,*N*,4-trimethylaniline
CAS number	99-97-8
Structural formula	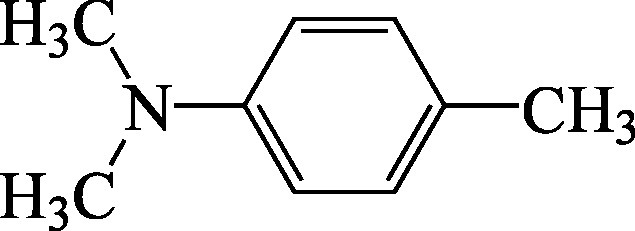
Molecular formula	C_9_H_13_N
Molar mass	135.21 g/mol
Melting point	–15 °C (ECHA 2020)
Boiling point at 1013 hPa	211 °C (ECHA 2020)
Density at 20 °C	0.94 g/cm^3^ (ECHA 2020)
Vapour pressure at 25 °C	0.237 hPa (NCBI 2022)
log K_OW_	2.81 (no other data); 1.729 (35 °C, pH 5.6); 2.61 (room temperature, pH > 7) (ECHA 2020)
Solubility at 25 °C	0.455 g/l water (ECHA 2020)
**1 ml/m^3^ (ppm) ≙ 5.61 mg/m^3^**	**1 mg/m^3^ ≙ 0.178 ml/m^3^ (ppm)**

Note: The substance can occur simultaneously as vapour and aerosol.

*N*,*N*-Dimethyl-*p*-toluidine is used as an initiator and accelerator in polymerization materials in dentistry and in bone cements. It is used for cross-linking in methyl methacrylates. In addition, it is a constituent of glues and artificial fingernails and is an intermediate in the production of dyes and pesticides (IARC 2018).

Dental resins contain 0.2% to 1.5% w/w of initiators relative to the monomer. Light activates camphorquinone, which decomposes to form a radical that is stabilized by *N*,*N*-dimethyl-*p*-toluidine. These initiators help to start the poly­merization reaction (Masuki et al. 2007; Noda et al. 2007).

*N*,*N*-Dimethyl-*p*-toluidine is present in all commercially used bone cements in concentrations of 0.7% to 2.6% (NTP 2012).

The European Union produces and imports 100 to 1000 t of *N*,*N*-dimethyl-*p*-toluidine each year. *N*,*N*-Dimethyl-*p*-toluidine was classified by registrants as not carcinogenic (“data conclusive but not sufficient for classification”) (ECHA 2020). The Committee for Risk Assessment classified the substance in Carc. Category 1B (RAC 2021).

NIOSH estimated that about 67 720 workers in the United States are potentially exposed to *N*,*N*-dimethyl-*p*-toluidine (NCBI 2020).

## 1 Toxic Effects and Mode of Action

*N*,*N*-Dimethyl-*p*-toluidine given by gavage to rats and mice for 2 years induced carcinogenic effects. Liver tumours were observed in both species. In addition, adenomas and carcinomas were found in the nasal transitional epithelium of male rats and in the lungs and forestomach of female rats.

Hyperplasia was induced in the transitional epithelium of the nose after only 5 days in male rats given 60 mg/kg body weight and day by gavage. In rats, gavage doses of 6 mg/kg body weight and day and above administered for 2 years led to increased methaemoglobin levels and effects on the spleen, liver and kidneys. In mice, effects on the liver were observed at this dose, but only female mice were found to have hyperplasia and metaplasia in the nose.

*N*,*N*-Dimethyl-*p*-toluidine caused irritation of the skin and eyes in rabbits.

Aneugenic, clastogenic and mutagenic effects were observed in mammalian cells in vitro. The incidences of DNA strand breaks in the liver were increased after *N*,*N*-dimethyl-*p*-toluidine was given to rats by oral administration and to mice by intraperitoneal injection.

There are only few positive findings for contact sensitizing effects of *N*,*N*-dimethyl-*p*-toluidine in humans. Findings for sensitization of the respiratory tract are not available.

There are no adequate data available for prenatal toxicity.

## 2 Mechanism of Action

*N*,*N*-Dimethyl-*p*-toluidine causes the toxic effects typical of aromatic amino compounds: methaemoglobin formation, weak genotoxicity and the development of liver tumours.

### 2.1 Methaemoglobin formation

Methaemoglobin formation is an event that occurs early after the administration of *N*,*N*-dimethyl-*p*-toluidine, and many effects such as anaemia and effects on the spleen result from the formation of methaemoglobin.

Accidental ingestion of *N*,*N*-dimethyl-*p*-toluidine induced methaemoglobin formation in children (Section 4.1). The oxidative effect was attributed to the putative metabolite *p*-methylphenylhydroxylamine (Dunnick et al. 2014). In a gavage study in rats, methaemoglobin values were already increased with statistical significance after 86 days at the lowest dose tested of 6 mg/kg body weight and day and above. In mice, methaemoglobin levels were increased only after the administration of 30 mg/kg body weight and day for 3 months. This is attributed to better detoxification due to the higher methaemoglobin reductase activity in this species (Section 5.2.2; NTP 2012).

The oxidative metabolite of *N*,*N*-dimethylaniline, phenylhydroxylamine, is one of the most potent methaemoglobin formers (Kao et al. 1997). Phenylhydroxylamine is the metabolite responsible for methaemoglobin formation also in the case of aniline (Hartwig 2010)*. *The corresponding metabolite of *N*,*N*-dimethyl-*p*-toluidine would be *p*-methylphenyl­hydroxylamine. As a result, *p*-methylphenylhydroxylamine is assumed to be the reactive intermediate compound in the metabolism of *N*,*N*-dimethyl-*p*-toluidine although it was not detected in the urine (IARC 2018; Kim et al. 2007).

In vitro studies with 0.1 mM *N*,*N*-dimethyl-*p*-toluidine or its metabolites *p*-methylnitrosobenzene and *p*-methylphenyl­hydroxylamine showed that methaemoglobin formation was induced only by the metabolites; after 1 hour, levels of 38.2% and 55.9% were determined in isolated erythrocytes. Likewise, aniline did not lead to methaemoglobin formation under the same conditions, whereas its metabolites nitrosobenzene and phenylhydroxylamine induced the formation of methaemoglobin levels of 13.7% and 48.4%, respectively. These values were somewhat lower than those of the corresponding metabolites of *N*,*N*-dimethyl-*p*-toluidine (Potter et al. 1988).

### 2.2 Reactive oxygen species (ROS)

The macrocytic, hypochromic and regenerative anaemia observed in rats given oral doses of *N*,*N*-dimethyl-*p*-toluidine is explained by haemoglobin damage caused by oxidative stress. The increased formation of methaemoglobin leads to irreversible haemichromes that precipitate in the form of Heinz bodies. This reduces erythrocyte deformability and accelerates the degradation of damaged erythrocytes in the spleen, thereby decreasing the number of erythrocytes. The damaged erythrocytes release redox-active iron, which may increase the formation of ROS via various processes. This mechanism is known from aniline and nitro aromatic compounds. In mice, the effects were less pronounced because of the higher methaemoglobin reductase activity or the lower rate of metabolism (Dunnick et al. 2014; Hartwig 2010).

In the human oral keratinocyte cell line OKF6/TERT2, ROS were increased with statistical significance after incubation with 1 or 2 mmol *N*,*N*-dimethyl-*p*-toluidine. This was determined by means of the oxidation-sensitive staining dye 2,7-dichlorofluorescein diacetate. The addition of 5 mM glutathione (GSH) markedly reduced the amount of ROS (Volk et al. 2014).

After incubation with 1 or 2 mM *N*,*N*-dimethyl-*p*-toluidine, ROS were detected in the Caco-2 human intestinal cell line by means of the oxidation-sensitive staining dye 2,7-dichlorofluorescein. The addition of *N*,*N*-dimethyl-*p*-toluidine caused a slight increase in ROS compared with the levels found in the control group, but the increase was statistically not significant (Wessels et al. 2015).

Administration of *N*,*N*-dimethyl-*p*-toluidine upregulated transcripts of the Nrf2 signalling pathway in the transitional epithelium of the nose. When the Nrf2 transcription factor binds to the antioxidative responsive element (ARE), it causes the transcription of various antioxidative enzymes and is regarded as a sensor of oxidative stress because ROS are able to induce the Nrf2 signalling pathway. In addition, 60 mg/kg body weight given by gavage to rats for 5 days induced effects on the nose, but did not lead to an increase in methaemoglobin levels. The authors assumed that this was due to low levels of antioxidative mucosubstances in the nasal cavity, which makes the nasal cavity susceptible to oxidative damage caused by *N*,*N*-dimethyl-*p*-toluidine (see Section 5.2.2; Dunnick et al. 2014). However, it is not known whether *N*,*N*-dimethyl-*p*-toluidine reaches the mucous because it acts systemically via the blood. In addition, intra­epithelial mucosubstances do not occur in the transitional epithelium (Harkema et al. 2006). Therefore, the explanation suggested by the authors that low levels of antioxidative mucosubstances in the transitional epithelium are responsible for the effects that were observed as early as after 5 days of treatment is not correct.

In isolated monocytes, 1 mM *N*,*N*-dimethyl-*p*-toluidine induced only slight changes in the GSH/glutathione disulfide (GSSG) ratio and the absolute GSH and GSSG levels. Thus, *N*,*N*-dimethyl-*p*-toluidine has no effects on the GSH redox balance in monocytes. In vitro, *N*,*N*-dimethyl-*p*-toluidine first began to inhibit succinate dehydrogenase activity with statistical significance at 1 mM; this effect increased with the concentration (Noda et al. 2007). It is assumed that the metabolism of *N*,*N*-dimethyl-*p*-toluidine is not activated in monocytes and no reaction is therefore expected.

### 2.3 Genotoxicity

Although aneugenic and clastogenic effects were observed in several in vitro genotoxicity tests and in a positive TK^+/–^ test, *N*,*N*-dimethyl-*p*-toluidine was not found to be mutagenic in the bacterial Salmonella typhimurium test even after metabolic activation. However, the mutagenic effects found in the TK^+/–^ test may have been caused by chromosomal aberrations. The high reactivity of the metabolites may be a possible explanation for the negative results that were obtained in the Salmonella typhimurium test. As a result, they may no longer be reactive by the time they reach the DNA. A test with Escherichia coli WP2 uvrA pKM101 yielded negative results. According to OECD Test Guideline 471, this test is recommended as an indicator of genotoxic effects via ROS (Section 5.6.1).

Likewise, structural analogues, such as *o*-toluidine and *p*-toluidine, did not clearly induce mutagenicity in the Salmonella typhimurium test. However, hydroxylamines and nitroso metabolites derived from *o*-toluidine and *p*-toluidine yielded positive results in this test system. This shows that these compounds develop mutagenic potential only if sufficient levels of metabolites have been formed by *N*-hydroxylation and subsequent *O*-acetylation (Greim 2005; Gupta et al. 1987).

As methaemoglobin formation was observed, it is likely that the reactive intermediate *p*-methylphenylhydroxyl­amine forms. This may yield a reactive quinone imine methide, an electrophile that may in turn form DNA adducts (Section 3.2; IARC 2018; Stepan et al. 2011).

**Fig.1:**
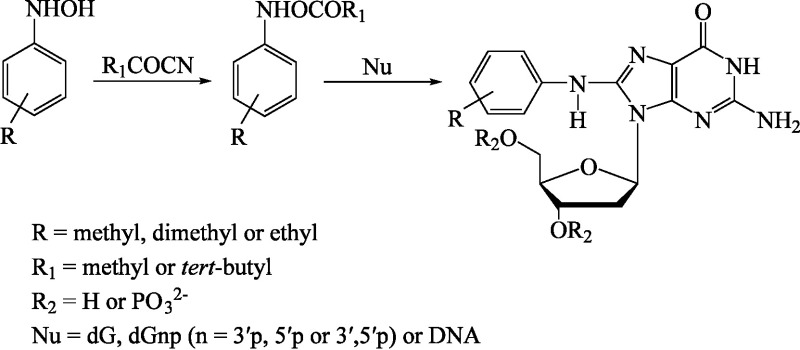
Synthesis of arylamine–DNA adducts (according to Marques et al. 1997)

*N*-Acyloxy arylamines (from methyl, dimethyl and ethyl anilines) that were generated in situ reacted with dG nucleo­tides and yielded an arylamine C8-dG adduct (see Figure 1). The DNA adducts were characterized by means of FAB mass spectrometry, UV spectrometry and H-NMR (Marques et al. 1997).

*N*,*N*-Dimethyl-*p*-toluidine and *N*-acyloxy arylamines are similar in structure. Therefore, *N*,*N*-dimethyl-*p*-toluidine may produce metabolites that form DNA adducts, leading then to genotoxic effects.

### 2.4 Carcinogenicity

The liver carcinomas observed in a 2-year gavage study in rats in the dose group given 60 mg/kg body weight were preceded by effects on the liver that had already developed at lower doses. At doses of 6 or 20 mg/kg body weight and day, pre-neoplastic precursors in the form of increases in eosinophilic foci, hyperplasia, hypertrophy and fibrosis were observed in the liver (Section 5.7; NTP 2012).

When rats were orally exposed to *N*,*N*-dimethyl-*p*-toluidine for 5 days, a transcriptomic analysis of the liver revealed the characteristic gene transcription patterns of a cell response to oxidative damage and liver lesions (Dunnick et al. 2017).

After 5-day oral exposure of rats to *N*,*N*-dimethyl-*p*-toluidine, a gene transcription pattern was found in the transitional epithelial cells of the nose that is characteristic of the cell response to oxidative damage, increased cell proliferation and a decrease in apoptosis signals (Dunnick et al. 2016).

In the 2-year gavage study, hyperplasia was observed in the lungs of mice in addition to a statistically significant increase in the incidence of adenomas. Effects ranging from degeneration, hyperplasia and metaplasia to adenomas and carcinomas were likewise observed in the nasal epithelium after oral administration of nitro aromatic compounds and acetaminophen. These effects were caused by metabolic activation in the nasal epithelium that is mediated by cytochrome P450 2E1, 2A5 and 2G1 (Genter et al. 1998; NTP 2012). Carcinogenic effects on the lungs may be more severe after inhalation exposure because of the irritant effects of *N*,*N*-dimethyl-*p*-toluidine.

The incidences of squamous cell papillomas were increased in the forestomach of female mice at the lowest dose tested of 6 mg/kg body weight and above. The authors suggested that these papillomas may have resulted from the gavage administration. However, although gavage was likewise used to administer corn oil to the animals of the vehicle control group, only 1 animal developed a forestomach papilloma. Therefore, it is more likely that the papillomas were the result of irritation induced by the substance immediately following administration of very high local concentrations rather than mechanical lesions (Section 5.3.1, 5.3.2; NTP 2012). Papillomas in the forestomach of rodents are not considered relevant to humans if they occur in isolated cases. However, if tumours are found in other organs, they can be regarded as further evidence of a carcinogenic potential (Laube et al. 2019).

Although the carcinogenic effects of aniline were found to have been caused by the formation of ROS following the production of methaemoglobin by damaged erythrocytes in the spleens of rats, the carcinogenicity of *N*,*N*-dimethyl-*p*-toluidine cannot be attributed solely to ROS formation. This is suggested by findings of neoplastic changes in the lungs and liver of mice after oral exposure to *N*,*N*-dimethyl-*p*-toluidine (Hartwig 2010). Although mice have higher levels of methaemoglobin reductase activity and thus form fewer ROS, a carcinogenicity study found adenomas and carcinomas at lower doses than in rats. This shows that another mechanism besides oxidative damage is responsible for tumourigenicity.

## 3 Toxicokinetics and Metabolism

### 3.1 Absorption, distribution, elimination

When groups of 4 male F344 rats were given ^14^C-labelled *N*,*N*-dimethyl-*p*-toluidine in single oral doses of 2.5, 25 and 250 mg/kg body weight (emulsion in 10% aqueous castor oil ethoxylate), 90.8%, 87.7% and 69.6% of the administered radio­activity was excreted with the urine and 3.4%, 9.3% and 1.8% was excreted with the faeces, respectively. Only negligible amounts were exhaled as CO_2_ and < 1% was exhaled as organic compounds. Twenty-four hours after the administration of 2.5 or 25 mg/kg body weight, 4.2% of the administered radioactivity was found in the body and gastro­intestinal tract. However, of the 18.3% recovered in the body after treatment with an oral dose of 250 mg/kg body weight, 12.3% was found in the gastrointestinal tract. This is an indication that the radioactivity had not been completely absorbed after 24 hours. Similar findings for absorption and excretion were obtained after oral exposure of male and female F344 rats to a dose of 25 mg/kg body weight. No differences were found with respect to absorption, distribution and excretion both after intravenous injection and oral administration of ^14^C-labelled *N*,*N*-dimethyl-*p*-toluidine in corn oil to male rats. About 100% of the radioactivity was recovered in the dose groups that were given 2.5 and 25 mg/kg body weight and about 90% in the high dose group. The tissue-to-blood partition coefficients were > 5 for the liver and kidneys 24 hours after administration of 2.5 mg/kg body weight. Administration of 250 mg/kg body weight in an aqueous solution led to a very high bladder-to-blood coefficient of about 15. The amounts excreted by male B6C3F1 mice after administration of 2.5 or 25 mg/kg body weight were similar to those excreted by rats. However, 24 hours after treatment with 250 mg/kg body weight, the mice were found in a moribund state with kidney damage, oedema in the lungs and changes in the gastrointestinal tract. The radioactivity recovered in the gastrointestinal tract and the rest of the body corresponded to 21.9% and about 10%, respectively, of the total amount administered. At this dose, 23.8% of the radioactivity was excreted with the urine and 8.0% with the faeces. The concentrations in the bladder and kidneys were 100 times higher than the levels determined after exposure to 25 mg/kg body weight and thus represented a disproportionate increase. In mice, about 85% to 100% of the radioactivity was recovered in the dose groups given 2.5 and 25 mg/kg body weight and about 65% in the high dose group. When mice were exposed to 25 mg/kg body weight, the tissue-to-blood partition coefficients were > 5 in the liver, about 5 in the kidneys, 8 in the bladder, 11 in the lungs and 7 in the adipose tissue (Dix et al. 2007). The acute toxicity of the substance impaired the data obtained at 250 mg/kg body weight because feed and water consumption were reduced at this dose.

There are no data available for the dermal absorption of *N*,*N*-dimethyl-*p*-toluidine. Using the IH SkinPerm model according to Tibaldi et al. (2014), it was calculated that 33 mg of substance would be absorbed after the exposure of 2000 cm^2^ of skin for 1 hour to a saturated aqueous solution of the substance. A much higher amount of 481 mg was estimated to be absorbed using the model of Fiserova-Bergerova et al. (1990). About the same levels of absorption were obtained for structurally related aromatic amines using model calculations (2,4-xylidine: 65 mg according to IH SkinPerm and 706 mg according to Fiserova-Bergerova et al. (1990); *p*-toluidine: 75 mg according to IH SkinPerm and 683 mg according to Fiserova-Bergerova et al. (1990)). These substances are designated with an “H” (for substances which can be absorbed through the skin in toxicologically relevant amounts) on the basis of their systemic toxicity. Therefore, *N*,*N*-dimethyl-*p*-toluidine is expected to be absorbed through the skin in relevant amounts.

### 3.2 Metabolism

**Fig.2:**
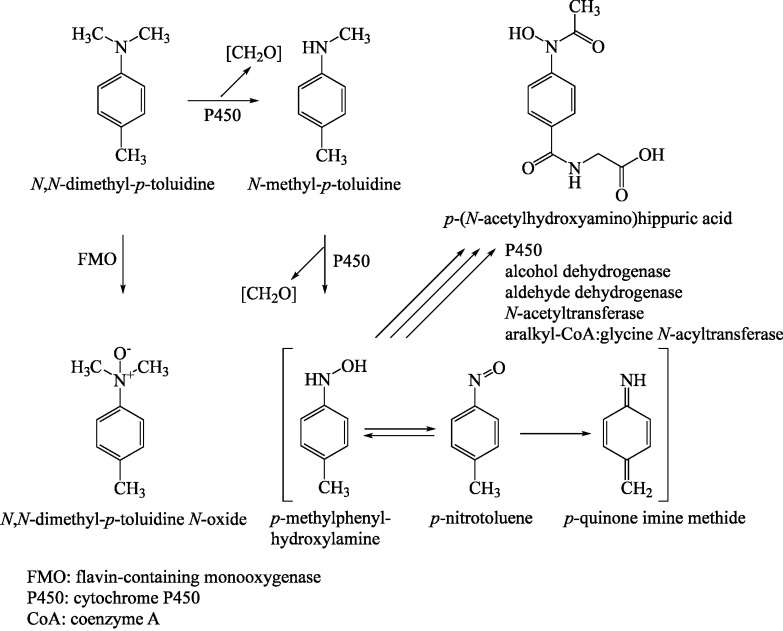
Extended metabolism of *N*,*N*-dimethyl-*p*-toluidine (according to IARC 2018)

After administration of ^14^C-*N*,*N*-dimethyl-*p*-toluidine to male F344 rats in single oral doses of 2.5 to 250 mg/kg body weight (in 10% aqueous castor oil ethoxylate), the metabolites in the urine were fractionated by HPLC. In addition to the parent substance, the major metabolite *p*-(*N*-acetylhydroxyamino)hippuric acid was identified, as well as *N*-methyl-*p*-toluidine and *N*,*N*-dimethyl-*p*-toluidine *N*-oxide as further metabolites. The metabolic pathway derived from these findings is shown in Figure 2. The metabolite *p*-methylphenylhydroxylamine that is responsible for methaemoglobin formation was not detected in the urine of male rats. However, the main metabolite *p*-(*N*-acetylhydroxyamino)hip­puric acid is the glycine conjugate of *N*-acetyl-*p*-methylphenylhydroxylamine. Therefore, it is assumed that *p*-methyl­phenylhydroxylamine forms in vivo from *N*,*N*-dimethyl-*p*-toluidine as a reactive intermediate (Section 2; IARC 2018; Kim et al. 2007).

Comparative studies of the analogous compound dimethylaniline in human and animal liver microsomes revealed that *N*-oxidation plays a more important role in humans, whereas *N*-demethylation is the main metabolic pathway in animals. In the rat liver, the formation of the *N*-oxide metabolite was catalysed by a flavin monooxygenase rather than by cytochrome P450 (Henschler 1993).

*N*-Demethylation and *N*-oxidation are pathways of *N*,*N*-dimethyl-*p*-toluidine that are mediated by cytochrome P450, with dealkylation being clearly favoured. The rate of dealkylation of *N*,*N*-dimethyl-*p*-toluidine catalysed by cyto­chrome P450 2B1 in vitro (Figure 2) was 39.8 ± 3.1 nmol/min/nmol cytochrome P450. This was much faster than oxidation at the nitrogen atom with a rate of 0.097 ± 0.017 nmol/min/nmol cytochrome P450; the ratio was 400 : 1 (Seto and Guengerich 1993). Therefore, the *N*-oxidation of *N*,*N*-dimethyl-*p*-toluidine is probably catalysed by flavin monooxygenase (Kim et al. 2007; Seto and Guengerich 1993).

The reactive hydroxylamine intermediate can be converted to an arylamine, which may be responsible for DNA adduct formation (Section 2.3).

## 4 Effects in Humans

### 4.1 Single exposures

A 5-month-old boy had a methaemoglobin level of 11% one hour after accidentally ingesting about 30 ml of a glue for artificial fingernails containing an unknown amount of *N*,*N*-dimethyl-*p*-toluidine. The child recovered after treatment with methylene blue and oxygen (IARC 2018; Kao et al. 1997).

A 16-month-old girl ingested 15 ml of a fingernail glue containing *N*,*N*-dimethyl-*p*-toluidine (80% acetone and 20% ethyl methacrylate monomer (98% with 2% *N*,*N*-dimethyl-*p*-toluidine = 60 mg)). After 2.5 hours, she developed cyanosis with a methaemoglobin level of 43%. The child recovered after administration of methylene blue (1 mg/kg body weight) (Potter et al. 1988)*.* The *N*,*N*-dimethyl-*p*-toluidine dose of 6 mg/kg body weight calculated by the authors is probably incorrect.

### 4.2 Repeated exposure

There are no data available.

### 4.3 Local effects on skin and mucous membranes

There are no data available.

### 4.4 Allergenic effects

#### 4.4.1 Sensitizing effects on the skin

The data available for the sensitizing effects of *N*,*N*-dimethyl-*p*-toluidine relate mainly to its use as a polymerization accelerator for dental and bone prostheses made of materials containing acrylate or methacrylate. Only very few reports attributed allergic contact dermatitis to occupational exposure to *N*,*N*-dimethyl-*p*-toluidine, for example as a constituent of such (meth)acrylate formulations. Most of the positive patch test results are associated with intolerance reactions to (dental) implants (Table 1).

For example, a commercially available 2% formulation of *N*,*N*-dimethyl-*p*-toluidine in petrolatum is included in the “bone cement components” test series of the German Contact Dermatitis Research Group (DKG). Tests conducted with this formulation in 9238 patients in the clinics of the Information Network of German Departments of Dermatology (Informationsverbund Dermatologischer Kliniken; IVDK) yielded a reaction index (defined as the quotient: (a−d−i) / (a+d+i); where: a = number of allergic reactions, d = number of questionable reactions and i = number of irritant reactions (Brasch and Henseler 1992)) of −0.73 and a positivity ratio (defined as the percentage of 1+ reactions among the total number of positive reactions (Geier et al. 2003)) of 100% (IVDK 2017). Therefore, testing with this formulation is problematical, and 1+ reactions may be false positives.

However, 5% formulations were used for testing in several studies from other European and non-European countries (Table 1). From 1987 to 1990 and 1991 to 1994, a total of 479 patients who had oral mucosal symptoms were patch tested with components of dental materials in the Department of Dermatology of Helsinki University Hospital. In the first period, no positive reactions were obtained with a 5% formulation of *N*,*N*-dimethyl-*p*-toluidine in petrolatum that was included in the test series. However, it is not clear how many patients were tested with this formulation and there is no information about possible questionable or irritant reactions (Alanko et al. 1996).

A review of the findings in health care workers that were recorded in the Finnish Register of Occupational Diseases between 2005 and 2016 reported one case of contact dermatitis caused by *N*,*N*-dimethyl-*p*-toluidine among 67 persons employed in the health care sector; no other details were included. Evidently, the remaining 197 health care workers who were tested (69 of them from the dental sector) did not produce reactions (Aalto-Korte et al. 2021).

**Tab.1:** Reported patch test results for *N*,*N*-dimethyl-*p*-toluidine in patients with suspected contact allergy

Persons tested	Test substance, concentration (vehicle)	Results	Contact / comments	References
**case reports**				
60-year-old joiner with early loosening of a hip prosthesis	no data	positive (palpable erythema after 48 and 96 hours)	no skin reactions after occupational exposure to numerous glues	Haddad et al. 1995
1 dental worker	2% (petrolatum)	positive (no other details)	patient with dermatitis 4 weeks after the beginning of exposure to dental materials	Rai et al. 2014
1 dental student with vesiculobullous changes on his fingertips	no data	positive (no other details)	additional positive reaction to a monomer liquid handled by the student (no other details)	Santosh et al. 1999
62-year-old housewife with diffuse redness and discomfort on the oral mucous membrane after receiving new dentures	1% (petrolatum)	1+ and 2+ (after 48 and 72 hours, respectively)	healing of the changes after the dentures had not been worn for a prolonged period	Tosti et al. 1990
79-year-old woman with discomfort (pain and burning) on the tongue several months after receiving new dentures	5% (petrolatum)	2+ (after 48 and 72 hours)	additional 2+ reaction to abrasion material of the prosthesis; no reaction to 5% *N*,*N*-dimethyl-*p*-toluidine in 139 other patients tested (no other details); symptoms reversible after a period without using the dentures	Verschueren and Bruynzeel 1991
**results of patch tests in larger cohorts**				
22 patients with burning mouth syndrome	5% (petrolatum)	positive in 3 of 22 (no other details)	test period not reported; 20 of the 22 patients with complete or partial dentures	Dutrée-Meulenberg et al. 1992
38 patients with implant intolerance	no data	positive in 0 of 38	test period not reported	Eben et al. 2010
725 patients	2% (petrolatum)	positive in 1 of 725 (no other details)	test period: 8/1992 to 7/1994; in addition, 10 × questionable and 1 × irritant reactions; overlapping of the cohort with that of Richter and Geier (1996)	Gebhardt and Geier 1996
40 dental technicians	2% (petrolatum)	positive in 0 of 40	test period: 1/1990 to 7/1993	Gebhardt et al. 1996
66 patients with planned total hip replacements, 14 patients with a stable hip prosthesis and 50 patients with loosening of their hip prosthesis	2% (petrolatum)	positive in 1 of 66, 0 of 14 and 2 of 50 (no other details)	test period: 1/2001 to 5/2004	Granchi et al. 2006
15 patients with early loosening of their hip prosthesis, 55 patients with total hip replacements	0.5% (no data)	positive in 7 of 15 and in 0 of 55 (no other details)	test period not reported	Haddad et al. 1996
53 dental workers with self-reported history of dermatitis on the hands	2% (petrolatum)	positive in 1 of 53 (1+, after 96 hours)	positive in a 42-year-old female orthodontist; relevance of the reaction not reported	Hill et al. 1998
43 patients	2% (petrolatum)	positive in 1 of 43 (no other details)	test period: 1981 to 1988; reaction was not considered to be relevant; testing with plastic and glue constituents	Holness and Nethercott 1997
9238 patients	2% (petrolatum)	positive in 9 of 9238 (1+, after 72 hours)	test period: 1997 to 2016; in addition 46 × questionable and 11 × irritant reactions; the 9 patients reacted on average to 6 different allergens	IVDK 2017
53 patients with burning mouth syndrome and dental prostheses	30% (olive oil)	positive in 1 of 53 (2+, after 48 or 72 hours)	positive in 1 patient with complete upper dentures; no reaction to test formulation in 20 control persons	Kaaber et al. 1979
143 patients	5% (petrolatum)	positive in 0 of 143	testing over a period of 3 years with plastic and glue constituents; possible overlapping of the cohort with that of Kanerva et al. (1999)	Kanerva et al. 1997
309 patients	5% (petrolatum)	positive in 0 of 309	test period: 1991 to 1996; testing with plastic and glue constituents; in addition, 1 × irritant reaction; possible overlapping of the cohort with that of Kanerva et al. (1997)	Kanerva et al. 1999
79 dentists and 46 dental assistants	5% (petrolatum)	positive in 0 of 125	test period: 1990 to 2000	Kiec-Swierczyńska and Krecisz 2002
150 dental technician students	5% (petrolatum)	positive in 9 of 150 (no other details)	positive reaction in 37 and 22 technicians to 2% formaldehyde and 5% *N*,*N*-bis(2-hydroxyethyl)-*p*-toluidine (in petrolatum), respectively	Lyapina et al. 2019
756 patients	2% (petrolatum)	positive in 1 of 756 (no other details)	test period: 1/1990 to 7/1993; overlapping of the cohort with that of Gebhardt and Geier (1996)	Richter and Geier 1996
35 dental technicians with occupational dermatitis on the hands	2% (petrolatum)	positive in 1 of 35 (no other details)	test period: 2/1993 to 6/1994; positive reaction in 1 of a total of 55 tested technicians; relevance unclear	Rustemeyer and Frosch 1996
33 patients with lichenoid lesions of the oral mucosa	2% (petrolatum)	positive in 1 of 33 (no other details)	test period: 2009 to 2012	Şahin et al. 2016
30 patients with lesions of the oral mucosa or lips	no data	positive in 0 of 30	test period: 1/1990 to 7/1998	Santosh et al. 1999
444 patients	5% (petrolatum)	0 of 444	test period: 2002 to 2007; irritant reaction in 1.6% of the patients; testing with plastic and glue constituents	Shmidt et al. 2010
115 patients with lichenoid lesions of the oral mucosa	2% (petrolatum)	positive in 2 of 115 (no other details)	test period: 11/2007 to 6/2014	Suter and Warnakulasuriya 2016
113 patients with implant intolerance	2% (petrolatum)	positive in 0 of 113	test period not reported	Thomas et al. 2008
250 patients with suspected allergy to bone implants	2% (petrolatum)	positive in 0 of 250	test period: 8/2010 to 9/2013	Thomas et al. 2015

#### 4.4.2 Sensitizing effects on the airways

There are no data available.

### 4.5 Reproductive and developmental toxicity

There are no data available.

### 4.6 Genotoxicity

There are no data available.

### 4.7 Carcinogenicity

There are no data available.

## 5 Animal Experiments and in vitro Studies

### 5.1 Acute toxicity

#### 5.1.1 Inhalation

When groups of 5 male and 5 female Sprague Dawley rats were exposed to *N*,*N*-dimethyl-*p*-toluidine aerosols in concentrations of 0, 300, 999, 1730 or 5270 mg/m^3^ and an MMAD of 0.23 to 0.57 µm for 4 hours, red material around the nose and nasal discharge were observed at concentrations of 300 mg/m^3^ and above. All animals of the highest concentration group and 60% of the animals exposed to 1730 mg/m^3^ died. An LC_50_ of 1400 mg/m^3^ was calculated from the data. Hypoactivity, loss of consciousness, dyspnoea or rapid breathing and increased salivation were observed in the animals exposed to 1730 mg/m^3^. Nasal discharge and dyspnoea were detected even in the animals exposed to 300 mg/m^3^ (ChemFirst Inc. 1997).

#### 5.1.2 Oral administration

The LD_50_ for mice was 139 mg/kg body weight. An LD_50_ of 980 mg/kg body weight was obtained for rats (ECHA 2020).

Groups of 5 male and 5 female Sprague Dawley rats were given single *N*,*N*-dimethyl-*p*-toluidine doses of 0, 1250, 1800 or 2500 mg/kg body weight. After 2 to 3 days, mortality was 1/5, 5/5 and 5/5 in the males and 1/5, 0/5 and 5/5 in the females, respectively. The LD_50_ was 1300 mg/kg body weight in male rats, 1950 mg/kg body weight in female rats and 1650 mg/kg body weight in both sexes combined. Other effects included hypoactivity, hunched posture and increased salivation (DuPont Haskell Global Centers for Health and Environmental Sciences 2009).

Single oral ^14^C-*N*,*N*-dimethyl-*p*-toluidine doses of 0, 2.5, 25 or 250 mg/kg body weight dissolved in water or corn oil were given to groups of 4 male F344 rats and 4 male B6C3F1 mice. Effects were observed only in the high dose group. Findings obtained in rats included reduced activity, piloerection, frequent eyelid closure, hunched posture and reduced feed and water consumption. No other clinical findings were detected after 24 hours. Laboured breathing was observed in mice 6 hours after administration and their activity levels and feed and water consumption were reduced. After 12 hours, the animals were lethargic and wheezed when breathing. One animal died and the other animals were found in a moribund state after 24 hours. The histopathological examination of the mice yielded light spots on the surface of the liver without any other effects on the organ, a distended gastrointestinal tract and mild oedema in the lungs. Accumulation of albuminous material, dilated tubules and sporadic vacuolation of the tubular epithelium were found in the kidneys. The methaemoglobin levels were not determined. At 2.5 mg/kg body weight and above, distinct discoloration, increased bilirubin values and blood were observed in the urine of treated mice after 12 to 24 hours; this is considered evidence of nephrotoxicity (Dix et al. 2007).

Six hours after gavage doses of 0, 600 or 1200 mg/kg body weight were given to groups of 5 male and 5 female Sprague Dawley rats, methaemoglobin levels of 12% to 13% were found in both sexes in the low dose group. After administration of 1200 mg/kg body weight, methaemoglobin levels of 30% and 13% were determined in the males and females, respectively. The methaemoglobin levels returned to the control values after observation for 7 days (DuPont Haskell Global Centers for Health and Environmental Sciences 2009).

#### 5.1.3 Dermal application

In rabbits, the LD_50_ after dermal absorption was higher than 2000 mg/kg body weight (US EPA 2009).

#### 5.1.4 Intraperitoneal and intravenous injection

The LD_50_ in mice was 212 mg/kg body weight after intraperitoneal injection (NTP 2012).

An LD_50_ value of 76 mg/kg body weight was obtained after intravenous injection into the tail veins of male SPF-NMR_1_ mice (Liso et al. 1997).

### 5.2 Subacute, subchronic and chronic toxicity

#### 5.2.1 Inhalation

There are no data available.

#### 5.2.2 Oral administration

The data from the studies reported below are shown in detail in Table 2.

When groups of 5 male F344/N rats were exposed 5 times to gavage doses of *N*,*N*-dimethyl-*p*-toluidine of 0, 1, 6, 20, 60 or 120 mg/kg body weight and day, the body weights were decreased at doses of 20 mg/kg body weight and day and above. Hyperplasia in the transitional epithelium of the nose, degeneration and necrosis of the olfactory epithelium in the nasal cavity and increased relative liver weights were observed in the two high dose groups. The methaemoglobin levels remained unchanged. The changes found in the tissue samples from the transitional epithelium of the nose in the high dose group included karyomegaly, compressed nuclei and the loss of basal polarity of the nuclei. Transcriptomic data of the nasal tissue revealed oxidative damage in the nasal transitional epithelium. In liver tissue samples, minimal lesions (increased incidence of apoptosis) were observed at doses of 20 mg/kg body weight and day and above and the rates of mitosis were slightly increased at 60 mg/kg body weight and day and above. The transcriptomic changes in the liver are also indicative of oxidative damage (Section 2.4; Dunnick et al. 2016, 2017).

In a 14-week gavage study, groups of 20 male and 20 female F344/N rats were given *N*,*N*-dimethyl-*p*-toluidine doses of 0, 62.5, 125, 250, 500 or 1000 mg/kg body weight and day (99.8%) dissolved in corn oil on 5 days a week. Haematological parameters were determined in half of the animals after 25 days. The animals of the high dose group and 1 male of the group that received 500 mg/kg body weight died within the first 3 days. The body weights were decreased with statistical significance at doses of 62.5 mg/kg body weight and day and above. Macrocytic, hypochromic and regenerative anaemia, methaemoglobinaemia and an increased formation of Heinz bodies were found in all animals including those of the low dose group. The effects increased in a dose-dependent manner and were observed as early as after 25 days. Cyanosis occurred in the high dose groups. At the low dose and above, the haematological effects induced lesions in the spleen (haematopoiesis, haemosiderin deposits, congestion and fibrosis) and bone marrow (hyperplasia). The alanine aminotransferase (ALT) and sorbitol dehydrogenase (SDH) activities were increased with statistical significance at the low dose and above; this increase was observed only on day 25, but not at the end of the subchronic study. The histopathological examination revealed increased liver weights, degeneration of the olfactory epithelium and metaplasia in the respiratory epithelium at the low dose and above. Hepatocellular hypertrophy, hyperplasia of Bowman’s glands and hyperplasia in the respiratory epithelium occurred at 125 mg/kg body weight and day and above. The incidences of metaplasia in the olfactory epithelium were increased with statistical significance at doses of 250 mg/kg body weight and day and above. The increased concentrations of bile acid in the serum at doses of 250 mg/kg body weight and day and above are indicative of liver damage and cholestasis (Dunnick et al. 2014; NTP 2012).

In a 2-year carcinogenicity study, groups of 60 male and 60 female F344/N rats were given gavage doses of *N*,*N*-di­methyl-*p*-toluidine of 0, 6, 20 or 60 mg/kg body weight and day on 5 days a week. The test substance had a purity of 99.8%. Haematological parameters were determined for 10 animals per group 3 months (86 days) after the beginning of treatment. In the females, Heinz bodies were increased dose-dependently with statistical significance even at 6 mg/kg body weight and day. The methaemoglobin levels were increased at 20 mg/kg body weight and day and above, and the body weights were slightly reduced at 60 mg/kg body weight and above. In the high dose group, increased mortality was associated with the occurrence of tumours, and neoplastic changes and hyperplasia of the olfactory, respiratory and transitional epithelium were observed in the nasal cavity (Section 5.7.2; Dunnick et al. 2014; NTP 2012). Haematological examinations were not carried out at the end of the 2-year oral treatment period although marked anaemia was observed after 3 months.

In a 14-week gavage study, groups of 10 male and 10 female B6C3F1 mice were treated with *N*,*N*-dimethyl-*p*-toluidine doses of 0, 15, 30, 60, 125 or 250 mg/kg body weight and day dissolved in corn oil on 5 days a week. The purity was determined to be 99.8%. In the high dose group, 9 males and all females died during the first 10 days. In the group that received 125 mg/kg body weight, 3 males and 2 females died before the end of the study. The body weights were decreased at 125 mg/kg body weight and day and above. The haematological examinations revealed slight decreases in the haematocrit values and erythrocyte counts, but they were not related to the dose. In the males, the methaemoglobin values increased minimally from 2.1% (control group) to 2.8% at 30 mg/kg body weight and day and above, at the higher doses to 3.1% and 4.0%. In the females, the values increased from 2.1% (control group) to 2.6%, 3.4% and 3.88%. Heinz body formation was increased with statistical significance in the females at 60 mg/kg body weight and day and above and in the males at 125 mg/kg body weight and day and above. At the low dose and above, the liver weights of the mice increased in a dose-related manner and with statistical significance. Degeneration and necrosis of the olfactory epithelium, hyperplasia of Bowman’s glands and degeneration of the bronchial epithelium in the lungs were observed in the dose group given 125 mg/kg body weight. Effects in the high dose group included fatty changes and necrosis in the liver, chronic active inflammation of the nose, and necrosis and atrophy in the lymph nodes, spleen and thymus. The authors attributed the effects observed in the high dose group to stress and non-specific toxicity (Dunnick et al. 2014; NTP 2012).

In a 2-year carcinogenicity study, groups of 50 male and 50 female B6C3F1 mice were given gavage doses of *N*,*N*-dimethyl-*p*-toluidine of 0, 6, 20 or 60 mg/kg body weight and day on 5 days a week. The purity was determined to be 99.8%. Effects on the liver occurred even in the low dose group. Effects on the lungs, nose and forestomach were observed at doses of 20 mg/kg body weight and day and above. The body weights were decreased in the high dose group. Haematological examinations were not carried out (Dunnick et al. 2014; NTP 2012).

**Tab.2:** Effects of *N*,*N*-dimethyl-*p*-toluidine after repeated oral administration

Species, strain, number per group	Dose	Findings	References
**rat**, F344, 5 ♂	**5 days**, 0, 1, 6, 20, 60, 120 mg/kg body weight and day, gavage, in corn oil	**20 mg/kg body weight and above**: body weights ↓, liver: incidence of apoptosis ↑; **60 mg/kg body weight and above**: liver: mitosis rate ↑, nose: transitional epithelium: hyperplasia (4/5); **120 mg/kg body weight**: liver: relative weights ↑*, nose: olfactory epithelium: necrosis (4/5), degeneration (1/5)	Dunnick et al. 2016, 2017
**rat**, F344, 10 ♂, 10 ♀	**25 days**, 0, 62.5, 125, 250, 500, 1000 mg/kg body weight and day, 5 days/week, gavage, in corn oil, haematological examination only	**0 mg/kg body weight**: MetHb ♂: 2.4%, ♀: 2.7%; **62.5 mg/kg body weight**: MetHb ♂: 6.7%, ♀: 6.4%; **62.5 mg/kg body weight and above**: body weights ↓*,anaemia, methaemoglobinaemia, haematocrit values ↓**, haemoglobin ↓**, erythrocytes ↓**, reticulocytes ↑**, ALT ↑*, SDH ↑*; **125 mg/kg body weight**: MetHb ♂: 12.44%, ♀: 12.8%; **125 mg/kg body weight and above**: Heinz bodies ↑**; **250 mg/kg body weight**: MetHb ♂: 16.6%, ♀: 16.0%; **500 mg/kg body weight**: MetHb ♂: 14.75%, ♀: 15.5%; **1000 mg/kg body weight**: mortality ♂ and ♀: 10/10 (after 3 days)	Dunnick et al. 2014; NTP 2012
**rat**, F344, 10 ♂, 10 ♀	**14 weeks**, MetHb determined after **88 days**; 0, 62.5, 125, 250, 500, 1000 mg/kg body weight and day, 5 days/week, gavage, in corn oil	**0 mg/kg body weight**: blood: MetHb ♂: 2.44%, ♀: 2.88%; **62.5 mg/kg body weight**: blood: MetHb ♂: 10.1%, ♀: 11.2%; **62.5 mg/kg body weight and above**: body weights ↓*, blood: anaemia, methaemoglobinaemia, haematocrit values ↓**, Heinz bodies ↑**, erythrocytes ↓**, reticulocytes ↑**, spleen: haematopoiesis ↑, haemosiderin deposition ↑, ♂: congestion ↑, bone marrow: hyperplasia*, haematopoiesis ↑, nose: olfactory epithelium: degeneration*, ♂: respiratory epithelium: metaplasia**, liver: relative weights ↑** (♂: 30%, ♀: 45%), kidneys: pigmentation*, relative weights ↑**, testis: relative weights ↑**, heart: relative weights ↑**; **125 mg/kg body weight**: MetHb ♂: 15.5%, ♀: 17.22%; **125 mg/kg body weight and above**: blood: serum: ♂: bile acid ↑*, spleen: congestion and capsular fibrosis**, ♂: hypertrophy**, nose: olfactory epithelium: degeneration**, respiratory epithelium: hyperplasia**, ♀: respiratory epithelium: metaplasia**, liver: weights ↑** (♂: 44%, ♀: about 64%), hypertrophy**, kidneys: ♂: necrosis and mineralization**, ♀: nephropathy**; **250 mg/kg body weight**: MetHb ♂: 18.2%, ♀: 19.7%; **250 mg/kg body weight and above**: blood: serum: ♂: bile acid ↑**, spleen: congestion and capsular fibrosis, hypertrophy**, nose: respiratory epithelium: hyperplasia**, olfactory epithelium: metaplasia**, liver: weights ↑** (♂: 57%, ♀: 89%), ♀: necrosis, kidneys: necrosis; **500 mg/kg body weight**: mortality ♂: 1/10 blood: MetHb ♂: 17.7%, ♀: 16.0%, serum: ♀: bile acid ↑**, liver: weights ↑** (♂: about 70%, ♀: about 123%), hypertrophy; **1000 mg/kg body weight**: mortality ♂ and ♀: 10/10 (after 3 days)	Dunnick et al. 2014; NTP 2012
**rat**, F344, 10 ♂, 10 ♀	**86 days**, 0, 6, 20, 60 mg/kg body weight and day, 5 days/week, gavage, in corn oil, haematological examination only	**0 mg/kg body weight**: MetHb ♂: 4.7%, ♀: 5.1%; **6 mg/kg body weight**: **LOAEL** MetHb ♂: 5.6%*, ♀: 5.6%, ♀: Heinz bodies ↑* (0.3%); **20 mg/kg body weight**: MetHb ♂: 7.9%**, ♀: 8.4%**, Heinz bodies ↑** (♂: 0.7%, ♀: 0.9%); **20 mg/kg body weight and above**: haematocrit values ↑**, erythrocytes ↓**, reticulocytes ↑**; **60 mg/kg body weight**: MetHb ♂: 17.4%**, ♀: 17.1%**, Heinz bodies ↑** (♂: 3.7%, ♀: 3.8%)	Dunnick et al. 2014; NTP 2012
**rat**, F344, 50 ♂, 50 ♀	**104 weeks**, 0, 6, 20, 60 mg/kg body weight and day, 5 days/week, gavage, in corn oil, no haematological examination	**6 mg/kg body weight and above**: **LOAEL** spleen: haematopoiesis ↑*, ♀: congestion and hypertrophy, ♂: atrophy*, liver: ♀: bile duct hyperplasia*, ♂: eosinophilic foci ↑*, kidneys: ♀: nephropathy*, ♂: pigmentation*, nose: glands, respiratory epithelium: metaplasia*, ♂: hyperplasia**, respiratory epithelium: ♂: hyperplasia**; **20 mg/kg body weight and above**: ♀: hyperactivity, liver: hypertrophy* (6/50), bile duct fibrosis**, ♀: eosinophilic foci ↑*, forestomach: ♂: hyperplasia and ulcer, bone marrow: ♂: hyperplasia*, lymph nodes: ♂: histiocytic infiltrates*, nose: glands, transitional epithelium: hyperplasia*, ♂: dilation*, respiratory epithelium: ♀: dilation**, hyperplasia**; **60 mg/kg body weight**: body weights ↓, mortality ↑ (caused by tumours), hyperactivity, ♀: pallor of the skin, spleen: congestion**, hypertrophy**, capsular fibrosis**, atrophy**, liver: hypertrophy ↑** (♂: 31/50, ♀: 22/49), cystic degeneration**, ♀: necrosis*, nose: lesions in the olfactory and respiratory epithelium*, kidneys: ♀: pigmentation*, forestomach: ♂: inflammation*, bone marrow: hyperplasia**	Dunnick et al. 2014; NTP 2012
**mouse**, B6C3F1, 10 ♂, 10 ♀	**14 weeks**,0, 15, 30, 60, 125, 250 mg/kg body weight and day, 5 days/week, gavage, in corn oil	**0 mg/kg body weight**: blood: MetHb ♂: 2.0%, ♀: 2.1%; **15 mg/kg body weight and above**: liver: weights ↑** (♂: 25.6%, ♀: 15.4%), vacuolation*; **30 mg/kg body weight and above**: blood: MetHb ↑ (♂: 2.8%**, ♀: 2.6%**); **60 mg/kg body weight and above**: blood: ♀: Heinz bodies ↑** (0.2%), nose: ♀: olfactory epithelium: degeneration*; **125 mg/kg body weight**: blood: Heinz bodies ↑ (♂: 0.5%**, ♀: 0.5%**), reticulocytes ♂: ↑*, nose: hyperplasia**, olfactory epithelium: degeneration**, metaplasia**, lungs: bronchial epithelium: degeneration* and regeneration*, peribronchial epithelium: chronic inflammation*, relative weights ↑**, ♀: histiocytic infiltrates*, thymus: necrosis*; **250 mg/kg body weight**: mortality ♂: 9/10, ♀: 10/10 (within 10 days)	Dunnick et al. 2014; NTP 2012
**mouse**, B6C3F1, 50 ♂, 50 ♀	**104 weeks**, 0, 6, 20, 60 mg/kg body weight and day, 5 days/week, gavage, in corn oil, no haematological examination	**6 mg/kg body weight and above**: liver: hypertrophy**, necrosis*, spleen: ♂: atrophy (11/50) not dose-dependent, bone marrow: ♀: hyperplasia* not dose-dependent, nose: ♀: hyperplasia in the olfactory epithelium**, metaplasia*; **20 mg/kg body weight and above**: spleen: ♂: atrophy* (11/49), liver: eosinophilic foci ↑*, lungs: ♀: hyperplasia, alveolar epithelium*, forestomach: ♀: hyperplasia**, nose: ♀: lesions in the olfactory and respiratory epithelium*; **60 mg/kg body weight**: ♀: body weights ↓ (> 10%), mortality ↑*, liver: ♀: fatty changes**, necrosis**, lungs: alveolar infiltrates (histiocytes)*, ♀: regeneration* (bronchial epithelium and bronchi), forestomach: ♀: inflammation and squamous cell papillomas**, nose: ♂: lesions in the olfactory and respiratory epithelium, spleen: ♀: atrophy of the red pulp*, lymph nodes: atrophy**	Dunnick et al. 2014; NTP 2012

* p < 0.05

** < 0.01

ALT: alanine aminotransferase; LOAEL: lowest observed adverse effect level; MetHb: methaemoglobin; SDH: sorbitol dehydrogenase

**Summary: **After 86 days, the methaemoglobin levels in rats were increased with statistical significance and Heinz bodies formed even at the low dose of 6 mg/kg body weight and day; effects on the spleen, liver and kidneys were increased after treatment for 2 years. Metaplasia and hyperplasia occurred in the nose. Hyperplasia of the transitional epithelium of the nose was likewise observed after administration of 60 mg/kg body weight and day for only 5 days. All effects increased in a dose-dependent manner. In addition, effects on the bone marrow, lymph nodes and forestomach were observed at higher doses.

In a 2-year gavage study, effects on the liver in male and female mice and hyperplasia and metaplasia of the nose in female mice were induced at the lowest dose tested of 6 mg/kg body weight and day. However, the slight effects on the spleen and bone marrow observed at this dose were not dose-related. At higher doses, the females had hyperplasia in the lungs and forestomach. In male mice, substance-induced effects on the nose and lungs were observed only at the high dose.

Therefore, the LOAEL (lowest observed adverse effect level) was 6 mg/kg body weight and day in rats and mice. A NOAEL (no observed adverse effect level) was not obtained.

#### 5.2.3 Dermal application

There are no data available.

### 5.3 Local effects on skin and mucous membranes

#### 5.3.1 Skin

When 0.5 ml of undiluted *N*,*N*-dimethyl-*p*-toluidine was applied to the shaved skin of 6 New Zealand White rabbits, erythema (score 1 to 2 of 3) was observed in all 6 animals at the end of the study after 14 days (DuPont Haskell Global Centers for Health and Environmental Sciences 2009). There are no other details of the type of application or its duration.

In a study carried out according to OECD Test Guideline 404, undiluted *N*,*N*-dimethyl-*p*-toluidine was applied semi-­occlusively to the intact shaved skin of 3 female New Zealand White rabbits for 4 hours. Erythema (score 1 to 2 of 3 according to Draize) was observed in only 1 rabbit 24 hours after application; however, this effect was no longer visible after 72 hours. The individual mean scores for erythema were 0.33 in 2 animals and 1.0 in 1 animal after 24, 48 and 72 hours. Oedema was found in only 1 animal up to a maximum of 24 hours after application and the individual score was 0.33 (ECHA 2020). Although the registration dossier reported that the coverage was “occlusive”, the description of the test procedure suggests a “semi-occlusive” type of application.

No corrosive potential (score 0.6 of 5) was found for *N*,*N*-dimethyl-*p*-toluidine in the dermal irritection model in vitro (according to OECD Test Guideline 435) (ChemFirst Inc. 1998). The OECD considers this model to be suitable for the identification of corrosive substances.

#### 5.3.2 Eyes

Undiluted *N*,*N*-dimethyl-*p*-toluidine (0.1 ml) was instilled into the right eye of 9 New Zealand White rabbits. The left eye of the animals was regarded as the control eye. After 30 seconds, the eyes of 3 animals were washed. Severe conjunctival redness (score 3 of 3) was observed in 1 animal of this group after 1 hour. The eyes of the other 6 animals were not washed. Severe conjunctival redness (score 3 of 3) was likewise found in one of these animals after 24 and 48 hours. All observed effects were reversible within 7 days (no other details; DuPont Haskell Global Centers for Health and Environmental Sciences 2009).

When 0.1 ml undiluted *N*,*N*-dimethyl-*p*-toluidine was instilled into the right eye of 3 female New Zealand White rabbits (washed after 1 hour) according to OECD Test Guideline 405, minimal irritation was observed only in the conjunctiva after 24 hours (score 0.67 of 3 according to Draize; individual values: 3 × 0.67 after 24, 48 and 72 hours). One animal had chemosis; the individual value was 0.33 (24, 48 and 72 hours). The effects were completely reversible within 72 hours after treatment (ECHA 2020).

In vitro, *N*,*N*-dimethyl-*p*-toluidine caused minimal to mild eye irritation (score: 13.2 of 80) in the ocular irritection model (according to OECD Test Guideline 496) (ChemFirst Inc. 1998). The OECD considers this model to be suitable for identifying severely irritant or non-irritant substances.

#### 5.3.3 Summary

In studies carried out according to OECD Test Guidelines 404 or 405, *N*,*N*-dimethyl-*p*-toluidine caused mild, reversible irritation of the skin and eyes in rabbits. However, these findings do not lead to classification of the substance according to CLP criteria.

### 5.4 Allergenic effects

There are no studies available that are relevant to the evaluation.

In a modified Buehler test, 50% and 10% *N*,*N*-dimethyl-*p*-toluidine in mineral oil was used for induction and challenge, respectively. The authors observed a skin reaction in 60% of the animals of the test group; this percentage was markedly higher than that found in the control group that was treated with the challenge concentration only (no other details; DuPont Haskell Global Centers for Health and Environmental Sciences 2009). The purity of the test substance was not reported, and there is no information about the number of animals per group or individual animal data. According to the authors, the incidence of reactions was much higher in the animals of the test group than in those of the control group; therefore, a reaction evidently occurred on the skin of some of the control animals as well. This study has not been included in the evaluation of the sensitizing potential of *N*,*N*-dimethyl-*p*-toluidine on the skin because of the lack of data and unclear findings.

### 5.5 Reproductive and developmental toxicity

#### 5.5.1 Fertility

Generation or fertility studies were not carried out.

The data available for the reproductive organs from 14-week and 104-week studies are reported below.

In a 14-week gavage study, groups of 10 male and 10 female B6C3F1 mice were given *N*,*N*-dimethyl-*p*-toluidine in doses of 0, 15, 30, 60, 125 or 250 mg/kg body weight and day dissolved in corn oil on 5 days a week. There were no effects on the oestrogen levels of the females or the weights of the reproductive organs. Likewise, no effects were determined for the sperm parameters (ECHA 2020; NTP 2012).

No effects were found on the reproductive organs in the 104-week gavage study in B6C3F1 mice up to the highest *N*,*N*-di­methyl-*p*-toluidine dose tested of 60 mg/kg body weight and day (Dunnick et al. 2014; NTP 2012).

In another 14-week gavage study, groups of 10 male and 10 female F344/N rats were given *N*,*N*-dimethyl-*p*-toluidine in doses of 0, 62.5, 125, 250, 500 or 1000 mg/kg body weight and day dissolved in corn oil on 5 days a week. No data were obtained from the animals of the two high dose groups because of the high mortality or the moribund state of the animals. In the dose groups that were given 125 and 250 mg/kg body weight, the number of females with regular oestrous cycles was reduced and the average length of the oestrus cycle of the animals was increased. Slight, but not statistically significant decreases in the sperm count were observed at doses of 125 mg/kg body weight and day and above. Sperm motility was not affected. The absolute, but not the relative testis and epididymis weights were reduced in the animals of the group that received 250 mg/kg body weight (Section 5.2.2; ECHA 2020; NTP 2012).

In the 104-week gavage study in F344 rats, histopathological effects on the reproductive organs were not observed up to the highest *N*,*N*-dimethyl-*p*-toluidine dose of 60 mg/kg body weight and day. The relative testis weights were increased with statistical significance at doses of 60 mg/kg body weight and day and above (Dunnick et al. 2014; NTP 2012).

#### 5.5.2 Developmental toxicity

There are no data available.

### 5.6 Genotoxicity

#### 5.6.1 In vitro

The data from the in vitro genotoxicity studies reported below are shown in detail in Table 3.

A gene mutation assay with Escherichia coli WP2 uvrA yielded negative results (NTP 2012).

*N*,*N*-Dimethyl-*p*-toluidine was not mutagenic in the Salmonella typhimurium strains TA97, TA98, TA100 or TA1535 either with or without the addition of metabolic activation (NTP 2012).

In the spot test, unclear genotoxicity in the form of a small increase in the number of revertants to 20–100 was detected after incubation of the Salmonella typhimurium strains TA100 and TA104 with an *N*,*N*-dimethyl-*p*-toluidine concentration of 3000 µg/plate. At the same concentration, gene mutations were not induced in the Salmonella typhimurium strain TA98 and cytotoxicity was detected in the Salmonella typhimurium strain TA97. However, *N*,*N*-dimethyl-*p*-toluidine concentrations of 0, 50, 250, 500, 2500 or 5000 µg/plate did not induce mutagenicity in the plate incorporation test in the Salmonella typhimurium strains TA97, TA98, TA100 and TA104 either with or without the addition of metabolic activation (Miller et al. 1986).

*N*,*N*-Dimethyl-*p*-toluidine was not mutagenic in the Salmonella typhimurium strains TA98, TA100, TA1535, T1537 or TA1538 either with or without the addition of metabolic activation (Seifried et al. 2006). The number of revertants was not included in the study report.

Mutagenicity was not found in another gene mutation test using the Salmonella typhimurium strains TA97, TA98 and TA100 and *N*,*N*-dimethyl-*p*-toluidine concentrations of 1 to 100 µg/plate. The purity of *N*,*N*-dimethyl-*p*-toluidine was 99% (Taningher et al. 1993).

*N*,*N*-Dimethyl-*p*-toluidine was not mutagenic in concentrations up to 5000 µg/plate in the Salmonella typhimurium strains TA98, TA100, TA1537 or TA1538 either with or without the addition of metabolic activation (US EPA 2009). The number of revertants was not reported.

Human intestinal adenocarcinoma cells (Caco-2 cells) are used as an in vitro model of the intestinal epithelium. After incubation with 1 or 2 mM *N*,*N*-dimethyl-*p*-toluidine, ROS were detected in the Caco-2 human intestinal cell line by means of the staining dye 2,7-dichlorofluorescein that is sensitive to oxidation. However, the increase in ROS was not statistically significant. A 2.5 molar concentration of *N*,*N*-dimethyl-*p*-toluidine did not affect the viability of the cells. After incubation in Caco-2 cells with 8-hydroxy-deoxyguanosine-DNA glycosylase 1 (hOGG1) in a modified comet assay, 2.5 mM *N*,*N*-dimethyl-*p*-toluidine led to a statistically significant increase in DNA damage and strand breaks of the type that develops after oxidative stress. Without the addition of hOGG1, DNA damage increased only slightly and the increase was not statistically significant (Wessels et al. 2015).

In a micronucleus test with V79 cells of Chinese hamsters, micronuclei were induced 48 hours after incubation with concentrations of 0.9 mM *N*,*N*-dimethyl-*p*-toluidine and above. Staining of the micronuclei with CREST antibodies revealed a statistically significant increase in both CREST-positive (at 0.9 mM and above) and CREST-negative micronuclei (at 12 mM). Therefore, clastogenic and aneugenic effects were induced. Cytotoxicity was investigated by colony-forming ability; the formation of colonies was still > 10% at the highest dose tested (no other details; Taningher et al. 1993). The mitotic index was investigated. However, the data do not clearly show whether dose-dependent, statistically significant changes were found.

In the TK^+/–^ mutation test carried out in mouse lymphoma cells according to OECD Test Guideline 490, the number of colonies were increased after incubation with *N*,*N*-dimethyl-*p*-toluidine concentrations of 0.031 µl/ml and above and metabolic activation; the relative total growth (RTG) was 12% to 15% (Seifried et al. 2006). The analysis did not differentiate between large colonies and small colonies. Therefore, no conclusions can be drawn as to whether mutagenic or clastogenic damage occurred.

**Tab.3:** Genotoxicity of *N*,*N*-dimethyl-*p*-toluidine in vitro

End point	Test system	Concentration range	Effective concentration	Cytotoxicity	Results		References
					–m. a.	+m. a.	
gene mutation	Escherichia coli WP2 uvrA pKM101	50–1500 µg/plate		500 µg/plate	–	–	NTP 2012
	Salmonella typhimurium TA100	10–1000 µg/plate		333/500 µg/plate	–	–^a)^	NTP 2012 (10 tests overall)
	Salmonella typhimurium TA1535	10–1000 µg/plate		500 µg/plate	–	–^a)^	NTP 2012 (8 tests overall)
	Salmonella typhimurium TA97	0.33–1000 µg/plate		500 µg/plate	–	–^a)^	NTP 2012 (9 tests overall)
	Salmonella typhimurium TA98	10–1000 μg/plate		333 µg/plate	–	–^a)^	NTP 2012 (10 tests overall)
	Salmonella typhimurium TA97 (spot test)	3000 μg/plate		3000 μg/plate	cytotoxic	cytotoxic	Miller et al. 1986
	Salmonella typhimurium TA98 (spot test)	3000 μg/plate			–	–	
	Salmonella typhimurium TA100 (spot test)	3000 μg/plate			(+)	(+)	
	Salmonella typhimurium TA104 (spot test)	3000 μg/plate			–	(+)	
	Salmonella typhimurium TA97, TA98, TA100, TA104 (plate incorporation test)	50–5000 µg/plate			–	–	
	Salmonella typhimurium TA98, TA100, TA1535, TA1537, TA1538 (plate incorporation test)	3–333 µg/plate			–	–	Seifried et al. 2006
	Salmonella typhimurium TA97, TA98, TA100	1–100 µg/plate		100 µg/plate	–	–^a)^	Taningher et al. 1993
	Salmonella typhimurium TA98, TA100, TA1537, TA1538 (plate incorporation test)	up to 5000 µg/plate		1000 μg/plate	–	–	US EPA 2009
	Salmonella typhimurium TA98, TA100, TA1537, TA1538	up to 100 µg/plate		100 µg/plate	–	–	
DNA damage by comet assay	Caco-2	2.5 mM	2.5 mM	–	(+) n. stat. sign.	n. t.	Wessels et al. 2015
DNA damage by comet assay (modified: hOGG1)	Caco-2	2.5 mM	2.5 mM	–	+*	n. t.	
micronucleus test (modified: 3–4 cell cycles)	V79 cells	0.3–1.2 mM	0.9 mM		+**	n. t.	Taningher et al. 1993
micronucleus test, CREST positive	V79 cells	0.3–1.2 mM	0.9 mM		+**	n. t.	
micronucleus test, CREST negative (CA)	V79 cells	0.3–1.2 mM	1.2 mM		+**	n. t.	
TK^+/–^ test	L5178Y mouse lymphoma cells	0.005-0.31 µl/ml	0.031 µg/ml	0.037 µg/ml	(+)	+	Seifried et al. 2006, 2008

^a)^ metabolic activation: S9 mix from rat or hamster liver

* < 0.05

** < 0.001 (Cochrane-Armitage trend test)

CA: chromosomal aberrations; CREST: kinetochore proteins; DMPT: *N*,*N*-dimethyl-*p*-toluidine; hOGG1: 8-hydroxy-deoxyguanosine-DNA glycosylase 1; n. stat. sign.: not statistically significant; n. t.: not tested

##### Mixtures

A genotoxicity test based on a supercoiled plasmid revealed an increased incidence of single strand breaks following incubation with a mixture of camphorquinone and *N*,*N*-dimethyl-*p*-toluidine (Lee et al. 2007; Winter et al. 2005). The frequency of micronuclei was increased significantly by a mixture of methyl acrylate, *N*,*N*-dimethyl-*p*-toluidine and hydroquinone in human lymphocytes (Bigatti et al. 1994), a mixture of camphorquinone and *N*,*N*-dimethyl-*p*-toluidine in CHO cells (Li et al. 2008) and a mixture of 9-fluorenone and *N*,*N*-dimethyl-*p*-toluidine in CHO cells (Li et al. 2007). Camphorquinone and *N*,*N*-dimethyl-*p*-toluidine are used together as initiators of the polymerization reaction, which proceeds via the formation of radicals. The DNA damage observed here can be explained by the expected formation of radicals. These studies are not suitable for evaluating *N*,*N*-dimethyl-*p*-toluidine as an individual substance.

#### 5.6.2 In vivo

A test for the induction of DNA strand breaks by alkaline elution revealed statistically significant increases in the elution rate and thus in DNA strand breaks in the liver in 4 male Sprague Dawley rats 6 hours after oral administration of an *N*,*N*-dimethyl-*p*-toluidine dose of 8 mmol/kg body weight (1081.7 mg/kg body weight) and 2 hours after intraperitoneal injection of 4 (3 animals) or 8 mmol/kg body weight (6 animals) (540.8 or 1081.7 mg/kg body weight). These effects were no longer observed after 24 hours. The control animals were divided into groups of 2 animals and the pooled controls were used for significance calculations. Male BALB/c mice were given intraperitoneal injections of *N*,*N*-dimethyl-*p*-toluidine doses of 0, 1 or 2 mmol/kg body weight (0, 135.2 or 270.4 mg/kg body weight). Although no effects were observed 2 hours after injection, a slight, but statistically significant increase in the formation of DNA strand breaks in the liver was found after 24 hours even at a dose of 1 mmol/kg body weight. The purity of the *N*,*N*-dimethyl-*p*-toluidine was 99%. The doses were established on the basis of LD_50_ values. However, the only value available for *N*,*N*-dimethyl-*p*-toluidine after intraperitoneal injection was that for mice of 212 mg/kg body weight (about 1.6 mmol/kg body weight) (Taningher et al. 1993). The LD_50_ after oral administration was 1300 mg/kg body weight (about 9.6 mmol/kg body weight) in male rats (Section 5.1.3; DuPont Haskell Global Centers for Health and Environmental Sciences 2009).

In the comet assay, groups of 6 Sprague Dawley rats were given *N*,*N*-dimethyl-*p*-toluidine in gavage doses of 0 or 60 mg/kg body weight and day for 4 days. A statistically significant increase in the percentage of tail DNA was found in the liver of the treated animals; the p value in Student’s t-test was p = 0.024 (NTP 2012).

Groups of 5 male B6C3F1/N mice were given *N*,*N*-dimethyl-*p*-toluidine in gavage doses of 0, 30, 60 or 75 mg/kg body weight and day in corn oil on 4 consecutive days. In the comet assay, no significant increase in DNA damage was observed in the leukocytes of the blood or liver (% tail DNA) 4 hours after the last dose. A test for increased micronucleus frequency in the peripheral blood, which was likewise sampled 4 hours after the last dose, yielded negative results. The number of reticulocytes remained unchanged (NTP 2012).

When *N*,*N*-dimethyl-*p*-toluidine was given to groups of 5 male and 5 female B6C3F1 mice in gavage doses of 0, 15, 30, 60 or 125 mg/kg body weight and day for 90 days (7 days/week), a small, but not statistically significant increase in the incidence of micronuclei was observed in the peripheral blood of the males at 60 mg/kg body weight and day and above. In this study, there were no changes in the number of reticulocytes in the circulating blood (NTP 2012). Therefore, it is unclear whether the substance reached the bone marrow.

#### 5.6.3 Summary

No mutations were observed in bacterial genotoxicity tests. However, the TK^+/–^ test in mouse lymphoma cells yielded positive results. *N*,*N*-Dimethyl-*p*-toluidine caused aneugenic and clastogenic effects in the micronucleus test in vitro. ROS are responsible for the DNA damage observed in the hOGG1-modified comet assay. In rats, increased incidences of DNA strand breaks in the liver were detected in the comet assay and by alkaline elution. In mice, the alkaline elution test revealed increased incidences of DNA strand breaks in the liver after intraperitoneal injection. However, no strand breaks were observed in the liver or peripheral blood after oral administration. When mice were given oral doses of *N*,*N*-dimethyl-*p*-toluidine for 4 days or 90 days, the number of micronuclei were not increased with statistical significance in the peripheral blood, and cytotoxic effects on the bone marrow were not observed up to the highest dose tested of 125 mg/kg body weight and day. Therefore, this study does not provide evidence that *N*,*N*-dimethyl-*p*-toluidine is absorbed into the bone marrow. In another 90-day study with oral administration, the number of reticulo­cytes in the circulating blood of the male mice was increased with statistical significance in the high dose group. In the 2-year carcinogenicity study, hyperplasia of the bone marrow was found in the females at doses of 6 mg/kg body weight and day and above; it is assumed that this is a secondary reaction of methaemoglobinaemia and the resulting oxidative stress rather than a cytotoxic effect on the bone marrow.

### 5.7 Carcinogenicity

#### 5.7.1 Short-term studies

There are no data available.

#### 5.7.2 Long-term studies

In a lifetime study, groups of 28 rats of the BDI, BDII and W strains were given *N*,*N*-dimethyl-*p*-toluidine doses of 0 or 7 mg/animal and day via their diet (about 21 mg/kg body weight and day; reported body weight: 300 g) until a total dose of 5 g/animal was reached. No tumours were detected. The lifespans and the body weight gains of the treated animals did not deviate from those of the control animals (Druckrey et al. 1954). The study does not comply with current guidelines and has therefore not been included in the evaluation.

In a 2-year study, groups of 50 male and 50 female F344/N rats were treated by gavage with *N*,*N*-dimethyl-*p*-toluidine doses of 0, 6, 20 or 60 mg/kg body weight and day on 5 days a week. The *N*,*N*-dimethyl-*p*-toluidine had a purity of 99.8% and was dissolved in corn oil. The vehicle control group was given only corn oil by gavage. In the high dose group, the incidence of hepatocellular carcinomas was increased with statistical significance in both sexes. A statistically significant increase in adenomas and carcinomas of the transitional epithelium was observed in the noses of the males in the high dose group. An adenoma was found in Bowman’s glands in the olfactory epithelium, which the authors considered unusual. The detailed data are shown in Table 4 and non-carcinogenic effects are described in Section 5.2.2 (NTP 2012).

In a 2-year carcinogenicity study, groups of 50 male and 50 female B6C3F1 mice were given gavage doses of *N*,*N*-dimethyl-*p*-toluidine of 0, 6, 20 or 60 mg/kg body weight and day on 5 days a week. *N*,*N*-Dimethyl-*p*-toluidine with a purity of 99.8% was dissolved in corn oil. The vehicle control group was given only corn oil by gavage. The incidences of hepatocellular carcinomas in the liver were increased with statistical significance in the females even in the low dose group and in the males of the high dose group. Squamous cell papillomas were observed in the forestomach of the females at the low dose and above. The authors suggested that the papillomas may have resulted from the use of the gavage route of administration. The incidences of bronchiolar-alveolar adenomas and carcinomas were increased in female mice at doses of 20 mg/kg body weight and day and above. However, only one carcinoma was found in each dose group. The data for carcinogenicity are shown in detail in Table 4 and 5 and non-carcinogenic effects are described in Section 5.2.2 (NTP 2012). It is not possible to explain the increase in the incidence of liver carcinomas in female B6C3F1 mice, which was found to be statistically significant even at low doses, based on the data available for metabolism. After exposure to *N*,*N*-dimethyl-*p*-toluidine, female mice evidently have a higher susceptibility to the development of these tumours than male mice, unlike in the case of many other substances. The squamous cell papillomas observed in the forestomach may have been substance-specific effects because *N*,*N*-dimethyl-*p*-toluidine induced metaplasia also in other organs. Therefore, they may not have been solely the result of irritation induced by the substance following the administration of very high local concentrations.

**Tab.4:** Carcinogenicity study with *N*,*N*-dimethyl-*p*-toluidine in rats

Author:	NTP 2012				
Substance:	*N*,*N*-dimethyl-*p*-toluidine (purity: > 99%)				
Species:	**rat**, F344/N, groups of 50 ♂, 50 ♀				
Administration route:	oral, gavage				
Dose:	0, 6, 20, 60 mg/kg body weight and day				
Duration:	2 years, 5 days/week				
Toxicity:	6 mg/kg body weight and day and above: liver: ♀: bile duct hyperplasia, ♂: eosinophilic foci ↑, spleen: haematopoiesis ↑, ♀: congestion and hypertrophy, nose: respiratory epithelium: metaplasia, ♂: hyperplasia, kidneys: ♀: nephropathy, ♂: pigmentation;20 mg/kg body weight and day and above: forestomach: ♂: hyperplasia and squamous cell papillomas, bone marrow: ♂: hyperplasia, lymph nodes: ♂: histiocytic infiltrates (Section 5.2.2)				
		**Dose [mg/kg body weight]**			
		**0**	**6**	**20**	**60**
surviving animals	♂	37/50 (74%)	37/50 (74%)	31/50 (62%)	21/50 (42%)*
	♀	33/50 (66%)	42/50 (84%)	33/50 (66%)	23/50 (46%)
**tumours and pre-neoplastic lesions**					
**liver**:					
hepatocellular carcinomas	♂	0/50	0/50	1/50 (2%)	6/50 (12%)*^a)^
	♀	0/50	0/50	0/50	4/49 (8%)*^b)^
hepatocellular adenomas and carcinomas	♂	0/50	0/50	2/50 (4%)	6/50 (12%)*
	♀	0/50	1/50 (2%)	1/50 (2%)	7/49 (14%)*
**nose**:					
adenomas, transitional epithelium	♂	0/50	3/49 (6%)	2/50 (4%)	11/49 (22%)*
	♀	0/50	1/49 (2%)	0/50	2/49 (4%)
carcinomas, transitional epithelium	♂	0/50	0/49	0/50	2/49 (4%)
adenomas and carcinomas, transitional epithelium	♂	0/50	3/49 (6%)	2/50 (4%)	13/49 (27%)*
**thyroid gland**:					
follicular adenomas	♂	1/50 (2%)	0/49	1/50 (2%)	3/49 (6%)
	♀	1/50 (2%)	1/47 (2%)	2/47 (4%)	0/45
follicular adenomas and carcinomas	♂	1/50 (2%)	2/49 (4%)	2/50 (4%)	4/49 (8%)
**uterus:**					
stromal polyps	♀	3/50 (6%)	9/50 (18%)	4/50 (8%)	8/50 (16%)

* p ≤ 0.05

^a)^ overall rate, poly-3 test p = 0.009

^b)^ overall rate, poly-3 test p = 0.041

**Tab.5:** Carcinogenicity study with *N*,*N*-dimethyl-*p*-toluidine in mice

Author:	NTP 2012				
Substance:	*N*,*N*-dimethyl-*p*-toluidine (purity: > 99%)				
Species:	**mouse, **B6C3F1, groups of 50 ♂, 50 ♀				
Administration route:	oral, gavage				
Dose:	0, 6, 20, 60 mg/kg body weight and day				
Duration:	2 years, 5 days/week				
Toxicity:	6 mg/kg body weight and day and above: liver: hypertrophy, necrosis, nose: ♀: hyperplasia, metaplasia; 20 mg/kg body weight and day and above: lungs: ♀: alveolar epithelium hyperplasia, forestomach: ♀: hyperplasia (Section 5.2.2)				
		**Dose [mg/kg body weight]**			
		**0**	**6**	**20**	**60**
surviving animals	♂	34/50 (68%)	36/50 (72%)	31/50 (62%)	36/50 (72%)
	♀	43/50 (86%)	40/50 (80%)	39/50 (78%)	32/50 (64%)
**tumours and pre-neoplastic lesions**					
**liver**:					
hepatocellular adenomas, multiple	♂	17/50 (34%)	19/50 (38%)	27/50 (54%)*	26/50 (52%)*
	♀	2/50 (4%)	6/50 (12%)	29/50 (58%)**	35/50 (70%)**
hepatocellular adenomas	♂	29/50 (58%)	34/50 (68%)	37/50 (74%)	36/50 (72%)
	♀	17/50 (34%)	19/50 (38%)	37/50 (74%)**	44/50 (88%)**
hepatocellular carcinomas	♂	22/50 (44%)	25/50 (50%)	30/50 (60%)	36/50 (72%)*
	♀	6/50 (12%)	13/50 (26%)*	18/50 (36%)**	31/50 (62%)**
hepatoblastomas	♂	1/50 (2%)	5/50 (10%)	10/50 (20%)*	8/50 (16%)*
	♀	0/50	1/50 (2%)	0/50	4/50 (8%)*
**lungs**:					
bronchiolar-alveolar adenomas	♀	2/50 (4%)	4/50 (8%)	8/50 (16%)*	12/50 (24%)*
bronchiolar-alveolar adenomas and carcinomas	♀	2/50 (4%)	5/50 (10%)	9/50 (18%)*	13/50 (26%)*
**forestomach:**					
squamous cell papillomas	♂	1/50 (2%)	1/50 (2%)	0/50	3/50 (6%)
	♀	1/50 (2%)	5/50 (10%)	6/50 (12%)*	7/50 (14%)*
squamous cell papillomas or carcinomas	♀	1/50 (2%)	6/50 (12%)	6/50 (12%)*	7/50 (14%)*

* p ≤ 0.05

** p ≤ 0.01

**Summary**: In male and female rats, the incidences of liver carcinomas and adenomas were increased with statistical significance after gavage doses of 60 mg/kg body weight and day were administered for 2 years. At this dose, the incidences of tumours in the transitional epithelium of the nose were increased with statistical significance in male rats.

After 2-year administration of gavage doses of *N*,*N*-dimethyl-*p*-toluidine, the incidences of liver carcinomas were increased with statistical significance in female mice even at a dose of 6 mg/kg body weight and day; in male mice, the increase was statistically significant only at doses of 60 mg/kg body weight and day and above. Hepatoblastomas were increased with statistical significance at doses of 20 mg/kg body weight and day and above in male mice and at the dose of 60 mg/kg body weight and day in female mice. Multiple hepatocellular adenomas in the liver were increased with statistical significance in both sexes at doses of 20 mg/kg body weight and day and above. The incidences of adenomas (and occasionally carcinomas) in the lungs and of squamous cell papillomas in the forestomach were increased with statistical significance in female mice at doses of 20 mg/kg body weight and day and above.

### 5.8 Other effects

The proliferation of human gingival fibroblasts was significantly reduced after incubation for 24 hours with *N*,*N*-dimethyl-*p*-toluidine concentrations of 500 µM and above. The cell cycle arrest in the G_0_/G_1_ phase increased with the dose. Staining with propidium iodide and annexin V showed that the cells were necrotic, but rarely apoptotic at concentrations of 1 mM *N*,*N*-dimethyl-*p*-toluidine and above (Masuki et al. 2007).

Various bone cements were fractionated by chromatography (HPLC) and the eluates containing *N*,*N*-dimethyl-*p*-toluidine were analysed for cytotoxicity after 15, 60 or 360 minutes. After incubation for 24 hours, the eluates containing *N*,*N*-dimethyl-*p*-toluidine inhibited the proliferation of the human osteoblast cell line MG63. A negative correlation was found between the *N*,*N*-dimethyl-*p*-toluidine concentration and the percentage of cells in the S-phase (Stea et al. 1997). The eluates did not contain only *N*,*N*-dimethyl-*p*-toluidine.

After incubation with 0 to 8 mmol *N*,*N*-dimethyl-*p*-toluidine, succinate dehydrogenase was inhibited in THP1 monocytes at concentrations of 1 mmol and above. After 4 hours, the GSH and GSSG levels were slightly increased at this *N*,*N*-dimethyl-*p*-toluidine concentration and above. *N*,*N*-Dimethyl-*p*-toluidine inhibited the NFκB transactivation induced by lipopolysaccharides in monocytes, whereas *N*,*N*-dimethyl-*p*-toluidine alone increased NFκB transactivation (Noda et al. 2007).

*N*,*N*-Dimethyl-*p*-toluidine induced a concentration-dependent increase in cytotoxicity (release of lactate dehydro­genase) in polymorphonuclear leukocytes from rats. The cytotoxicity was determined by comparing the lysis ability of the substance with that of Triton X-100. The minimum inhibition concentration in bacteria was 185 mM (Liso et al. 1997).

*N*,*N*-Dimethyl-*p*-toluidine displayed oestrogen antagonist activity in a reporter gene assay (yeast two-hybrid system) with metabolic activation (Nomura et al. 2003).

Cytotoxicity was not increased in cultured human oral keratinocytes OKF6/TERT2 after incubation with 0.1 to 2.5 mmol *N*,*N*-dimethyl-*p*-toluidine for 24 or 48 hours. The increase in apoptosis induced by camphorquinone was not affected by *N*,*N*-dimethyl-*p*-toluidine (Volk et al. 2014).

## 6 Manifesto (MAK value/classification)

The critical effects are methaemoglobinaemia in humans and animals, and anaemia and hepatotoxicity in rodents. Carcinogenicity was observed in rats and mice.

**Carcinogenicity. **In a 2-year gavage study, the incidence of carcinomas in the liver was increased with statistical significance in female B6C3F1 mice at doses of 6 mg/kg body weight and day and above. After administration of 20 mg/kg body weight and day, the incidence of hepatoblastomas in male mice and the incidence of adenomas (and carcinomas) in the lungs of female mice were increased with statistical significance. Although B6C3F1 mice are known to have high spontaneous incidences of liver tumours, hepatoblastomas are rarely found in B6C3F1 mice (with a low incidence of 4% in the historical control data) (NTP 2012). They are thus evidence of carcinogenic effects.

In F344 rats, the incidences of adenomas and carcinomas in the liver were increased with statistical significance after the administration of gavage doses of 60 mg/kg body weight and day for 2 years. In this dose group, adenomas and carcinomas were found also in the transitional epithelium of the nose in the male rats. Neoplasms in the transitional epithelium of the nose were not reported in the historical control data obtained from NTP studies (NTP 2012). Therefore, they are regarded as substance-induced carcinogenic effects.

Squamous cell papillomas developed in the forestomach of female mice. They may be the result of irritation induced by high local concentrations of the substance after administration by gavage.

The absorption of *N*,*N*-dimethyl-*p*-toluidine is assumed to lead to metabolites that induce the formation of DNA adducts. Together with ROS, these adducts may be responsible for the genotoxic effects (Marques et al. 1997).

*N*,*N*-Dimethyl-*p*-toluidine has been classified in Carcinogen Category 2 because tumours were observed in different species (rats and mice), in both sexes and in various organs.

**MAK value and peak limitation. **It was not possible to derive a NOAEC (no observed adverse effect concentration) for carcinogenicity based on the data available from animal studies. Genotoxicity was detected and was probably partially responsible for the carcinogenic effects.

Therefore, a MAK value has not been derived and a peak limitation does not apply.

**Prenatal toxicity. **There are no data available for developmental toxicity. As no MAK value was derived, *N*,*N*-di­methyl-*p*-toluidine has not been classified in any of the pregnancy risk groups.

**Germ cell mutagenicity. ***N*,*N*-Dimethyl-*p*-toluidine caused DNA damage in mammalian cells in vitro in a modified comet assay, and aneugenic and clastogenic effects were detected in a micronucleus test. No mutations were observed in bacterial genotoxicity tests, but a TK^+/–^ test yielded positive results. An analysis by alkaline elution showed increased incidences of DNA strand breaks in the livers of rats and mice. A comet assay with oral exposure found that the incidence of DNA strand breaks in the liver was increased with statistical significance in rats, but not in mice. The incidences of micronuclei were not increased with statistical significance in the peripheral blood of mice after oral administration for 4 or 90 days; however, it is unclear whether the bone marrow was reached. There are no studies available that investigated germ cells. *N*,*N*-Dimethyl-*p*-toluidine has been classified in Category 3 B because no studies have become available that provide evidence contradicting the increased incidence of DNA strand breaks in rats and mice.

**Absorption through the skin. **There are no data available for the absorption of *N*,*N*-dimethyl-*p*-toluidine through the skin. However, the results of model calculations suggest that *N*,*N*-dimethyl-*p*-toluidine is absorbed through the skin in relevant amounts. As the substance has been classified as genotoxic in Carcinogen Category 2, *N*,*N*-dimethyl-*p*-toluidine has been designated with an “H” (for substances which can be absorbed through the skin in toxicologically relevant amounts).

**Sensitization. **There are only few findings relating to the contact sensitizing effects of *N*,*N*-dimethyl-*p*-toluidine. According to these, *N*,*N*-dimethyl-*p*-toluidine does not cause strong contact allergy. No findings are available for sensitizing effects on the airways. As a result, *N*,*N*-dimethyl-*p*-toluidine has not been designated with either “Sh” or “Sa” (for substances which cause sensitization of the skin or airways).
